# Development of a predictive model for adverse outcomes after unilateral biportal endoscopic spine surgery in the treatment of lumbar disc herniation using six interpretable machine learning models

**DOI:** 10.3389/fsurg.2026.1908118

**Published:** 2026-08-31

**Authors:** Yuelong Tan, Yu Xi, Naiyan Hu, Lin Liu, Luo Xu

**Affiliations:** 1 Department of Orthopedics, Ansteel General Hospital, Anshan, Liaoning, China; 2 Journal Center of China Medical University, Shenyang, Liaoning, China

**Keywords:** explainable artificial intelligence, lumbar disc herniation, machine learning, predictive model, unilateral biportal endoscopic spine surgery

## Abstract

**Objective:**

This study aimed to develop and validate an interpretable machine learning model incorporating clinical and radiological features to predict the risk of adverse outcomes in patients with lumbar disc herniation (LDH) following unilateral biportal endoscopic (UBE) surgery. The model was designed to facilitate preoperative risk stratification and assist individualized clinical decision-making.

**Methods:**

A retrospective cohort study was conducted involving 418 patients with LDH who underwent UBE surgery at our institution between January 2022 and January 2025. Patients were randomly allocated to the training and validation cohorts in a 7:3 ratio. The collected variables comprised demographic characteristics, perioperative parameters, and radiological features. Four complementary feature selection approaches, including the least absolute shrinkage and selection operator (LASSO), Boruta, minimum redundancy maximum relevance (mRMR), and recursive feature elimination (RFE), were applied. Six machine learning algorithms were developed and optimized using five-fold cross-validation within the training cohort. The predictive performance of each model was subsequently evaluated in the validation cohort, and the area under the receiver operating characteristic curve (AUC) was used to identify the optimal model. The best-performing model was further evaluated using multiple performance metrics. SHapley additive explanation (SHAP) analysis was performed to interpret model predictions and enhance transparency in the decision-making process. Finally, an online risk prediction calculator was developed based on the optimal model to facilitate clinical application.

**Results:**

Modic changes, Pfirrmann grade, APHC, and age were identified as important predictors of adverse outcomes following UBE surgery. Among the six evaluated machine learning models, the ExtraTrees model demonstrated the highest predictive performance. In the training cohort, the model achieved an AUC of 0.933 (95% CI, 0.901–0.960), with an accuracy, precision, sensitivity, and specificity of 0.850, 0.867, 0.703, and 0.934, respectively. In the validation cohort, the model maintained satisfactory performance, achieving an AUC of 0.884 (95% CI, 0.818–0.941), with an accuracy, precision, sensitivity, and specificity of 0.818, 0.811, 0.652, and 0.913, respectively, suggesting good generalizability. Decision curve analysis demonstrated that the ExtraTrees model achieved greater net clinical benefit than the other models across a wide range of threshold probabilities. SHAP-based global importance plots, dependence plots, and individual-level waterfall and force plots demonstrated that higher Modic grades, higher Pfirrmann grades, increased APHC values, and older age were associated with an increased risk of adverse outcomes. The direction and magnitude of these associations were largely consistent with clinical expectations, thereby enhancing model interpretability and clinical acceptance. Furthermore, a web-based calculator (https://mtt123456.shinyapps.io/dynnomapp/) was developed to facilitate the practical application of the prediction model.

**Conclusion:**

The proposed prediction model demonstrated favorable predictive performance in the internal validation cohort and may serve as a practical tool for preoperative risk assessment of adverse outcomes following UBE surgery for LDH. This model may assist in identifying high-risk patients and provide evidence to support preoperative planning, patient counseling, and individualized treatment optimization. However, because validation was limited to a randomly divided single-center dataset, further external validation involving multicenter cohorts, different temporal periods, and surgeons with varying levels of experience is required to determine the model's robustness and generalizability.

## 1 Introduction

In recent years, advances in minimally invasive spinal surgery have expanded its application in the management of lumbar disc herniation (LDH). Owing to advantages including reduced intraoperative blood loss, shorter hospital stays, and less tissue trauma, minimally invasive techniques have gained increasing attention among orthopedic surgeons. Among these techniques, unilateral biportal endoscopic (UBE) surgery has emerged as a representative minimally invasive approach and has been increasingly adopted for LDH surgery (1). However, UBE is a technically demanding procedure characterized by a steep learning curve, requiring surgeons to possess advanced skills in endoscopic anatomical recognition, coordinated handling of dual-channel instruments, maintenance of an adequate operative field, and precise control of decompression extent (2, 3). In clinical practice, adverse outcomes following UBE may result from multiple factors, including unstable instrument handling, inadequate visualization, insufficient anatomical recognition, inappropriate indication selection, and suboptimal preoperative assessment or surgical workflow standardization (4). Therefore, establishing a reliable preoperative risk prediction model is clinically important for identifying patients at high risk of adverse outcomes after UBE for LDH, optimizing patient selection, and developing individualized treatment strategies.

Previous studies have developed nomogram-based models to predict adverse outcomes after minimally invasive spinal surgery. However, conventional statistical methods typically rely on predefined linear assumptions and have limited ability to capture complex interactions and nonlinear relationships among variables. When postoperative outcomes are influenced by multiple clinical and radiological factors, traditional models may fail to fully capture underlying patterns within complex datasets, thereby limiting predictive performance (5). In recent years, machine learning (ML) has gained increasing attention in medicine and has been increasingly applied to predict postoperative complications and clinical outcomes (6–8). Compared with conventional statistical approaches, ML algorithms are better suited to handling complex data structures, nonlinear relationships, and interactions among multiple variables, making them particularly suitable for multifactorial clinical prediction tasks (9, 10). However, the complexity of ML models may compromise interpretability, representing a major barrier to their clinical implementation. To address this limitation, explainable artificial intelligence approaches have been increasingly explored to improve the interpretability of machine learning models. Among these methods, Local Interpretable Model-agnostic Explanations (LIME) and SHapley Additive exPlanations (SHAP) are two widely used approaches. LIME explains individual predictions by constructing local surrogate models, enabling assessment of the contribution of specific features to model outputs in individual cases. Based on Shapley value theory, SHAP quantifies the contribution of individual features to model predictions and provides visual explanations at both local and global levels, thereby helping to overcome the “black-box” limitation of machine learning models (11, 12). Furthermore, SHAP provides consistent and stable interpretations, which may improve model transparency and clinical acceptance (13). Therefore, this study aimed to develop and validate an interpretable machine learning model incorporating clinical and radiological characteristics to predict adverse outcomes following UBE surgery in patients with LDH. The proposed model was designed to facilitate preoperative risk stratification and identify high-risk patients, thereby supporting patient selection, preoperative counseling, and perioperative management. Furthermore, LIME and SHAP analyses were performed to evaluate the interpretability of the optimal model. An online risk prediction calculator was also developed to provide an accessible decision-support tool for individualized clinical decision-making in patients undergoing UBE surgery for LDH.

## 2 Methods

### 2.1 Patient selection

**Figure 1:**
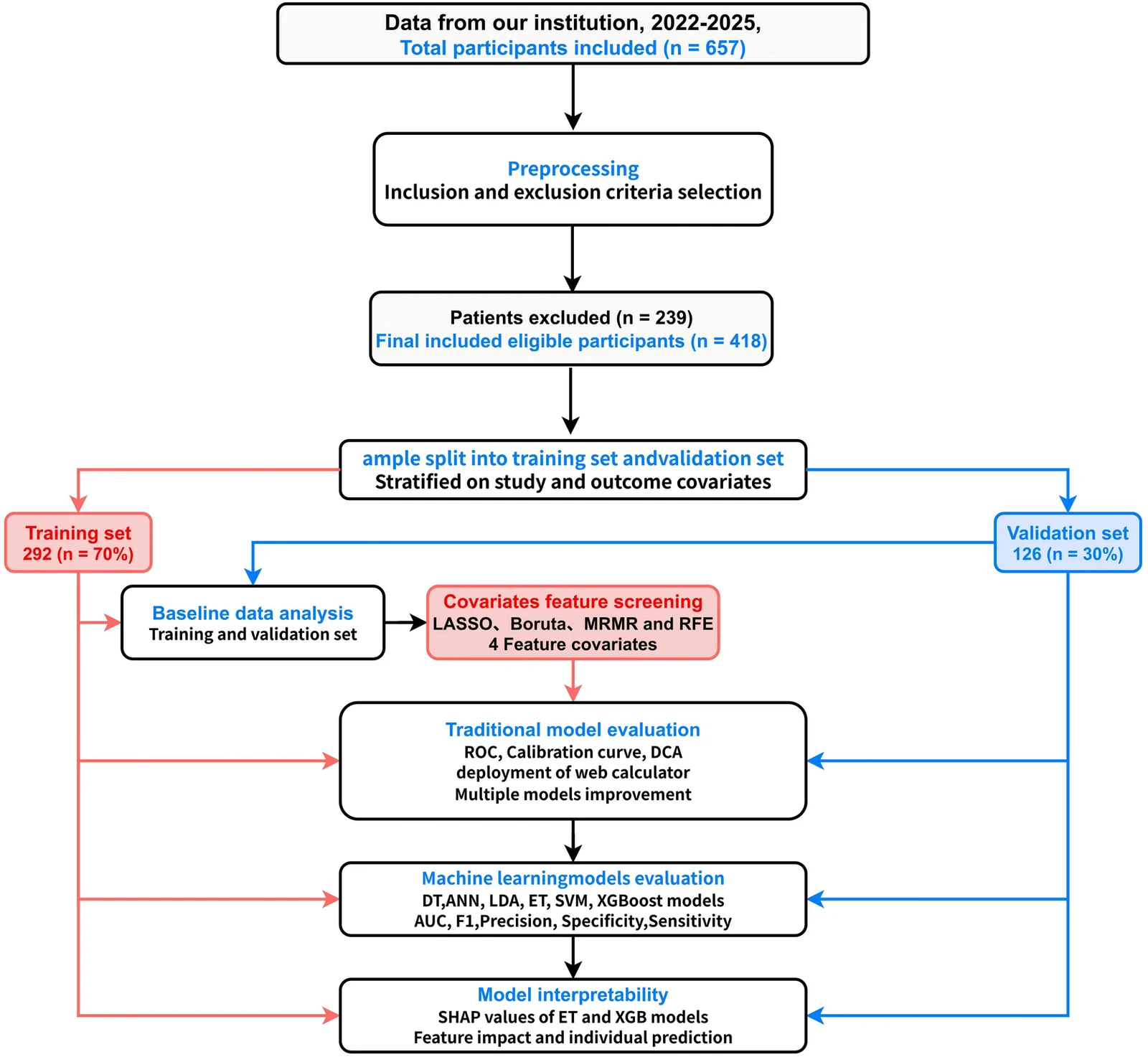
Flowchart depicts the patient's enrollment process in the study. ANN, artificial neural network; AUC, area under the receiver operating characteristic curve; DCA, decision curve analysis; DT, decision tree; ET, extremely randomized trees; F1, F1 score; LASSO, least absolute shrinkage and selection operator; LDA, linear discriminant analysis; mRMR, minimum redundancy–maximum relevance; RFE, recursive feature elimination; ROC, receiver operating characteristic; SHAP, Shapley additive explanations; SVM, support vector machine; XGB, extreme gradient boosting.

Clinical data from 418 patients with lumbar disc herniation who underwent UBE surgery at our institution between January 2022 and January 2025 were retrospectively reviewed. All procedures were performed by a single spine surgeon with extensive experience in minimally invasive lumbar decompression. This approach reduced inter-surgeon variability in surgical technique and operator experience. Patients were classified into favorable- and adverse-outcome group according to their postoperative outcomes. The cohort was randomly divided into a training set and a validation set in a 7:3 ratio, yielding 292 and 126 patients, respectively. A fixed random seed of 123 was used to ensure that the data-partitioning procedure was reproducible. After data partitioning, the proportions of adverse outcome were 38.0% in the training set and 36.5% in the validation set, indicating similar outcome prevalence in the two sets. Following data partitioning, all preprocessing, feature selection, and model hyperparameter tuning were performed exclusively within the training set. The validation set was kept separate throughout model development and was used only for the final evaluation of model performance, thereby minimizing the risk of information leakage. The study protocol was approved by the institutional ethics committee (approval no. 2026-01), and the study was conducted in accordance with the Declaration of Helsinki. The inclusion criteria were as follows: (1) concordance between clinical symptoms and signs and the imaging findings; (2) lumbar spine surgery performed for the first time; and (3) availability of complete clinical and imaging data. The exclusion criteria were as follows: (1) a history of lumbar spine surgery or contraindications to surgery or anesthesia; (2) instability at the index level or concomitant lumbar spinal stenosis; (3) a history of revision surgery at the same index level; (4) severe cardiac, hepatic, or renal dysfunction; (5) other spinal disorders, including ankylosing spondylitis, spinal tumors, spinal fractures, and spinal tuberculosis; and (6) incomplete clinical or imaging data or loss to follow-up. The study workflow is presented in Figure 1.

### 2.2 Model development, evaluation, and interpretation

Potential predictors were initially identified through a comprehensive literature review of variables previously reported to be associated with postoperative outcomes. Candidate variables were subsequently selected based on available evidence and expert consultation with spine surgeons (14). A total of 17 candidate variables were included in the analysis. All variables were systematically collected from the electronic health record (EHR) system and preoperative imaging data, including demographic characteristics, perioperative parameters, and radiological features. Demographic and perioperative variables included age, sex, body mass index (BMI), alcohol consumption, smoking status, diabetes mellitus, hypertension, duration of disease (COD), operative time (OT), American Society of Anesthesiologists (ASA) classification, and osteoporosis status. Radiological variables included disc calcification, Pfirrmann grade (PG), Modic changes (MC), disc herniation level, herniation type (HT), and facet joint hypertrophy and bony fusion (APHC). To reduce observer bias in radiological assessment, all preoperative lumbar magnetic resonance imaging (MRI) and computed tomography (CT) images were independently reviewed by two physicians experienced in spinal imaging interpretation. Both reviewers were blinded to postoperative outcomes and model predictions throughout the assessment process. Modic changes were assessed using preoperative lumbar T1- and T2-weighted MRI images and classified as absent, type I, type II, or type III. Pfirrmann grading was used to evaluate intervertebral disc degeneration based on preoperative sagittal T2-weighted lumbar MRI images and classified degeneration from grade I to grade V.APHC was assessed based on preoperative lumbar CT and MRI findings and defined as prominent facet joint hypertrophy, osteophyte formation, or bony fusion-like changes at the target segment, accompanied by narrowing of the lateral recess, intervertebral foramen, or endoscopic working channel. When discrepancies occurred between the two reviewers, the images were jointly reviewed and discussed. If disagreement persisted, the final decision was made by a senior spine surgeon. Interobserver agreement for radiological assessments was evaluated using the kappa coefficient. The primary outcome was the 3-month postoperative clinical outcome, which was determined according to the modified MacNab criteria, visual analog scale (VAS) scores, and postoperative events. A favorable outcome was defined as a modified MacNab rating of excellent or good, a VAS score <4 at 3 months postoperatively, and no postoperative events, including symptom aggravation, incomplete symptom relief, or recurrence. An adverse outcome was defined as a modified MacNab rating of fair or poor, a VAS score ≥4, or the occurrence of any postoperative event, including symptom aggravation, incomplete symptom relief, or recurrence. For patients with inconsistent findings across evaluation criteria, fulfillment of any adverse outcome criterion resulted in classification into the adverse outcome group. After dataset partitioning, all feature selection procedures and model optimization were performed only in the training cohort to prevent information leakage and ensure reliable model evaluation. To improve the robustness of feature selection, four complementary approaches were applied: least absolute shrinkage and selection operator (LASSO) regression, the Boruta algorithm, minimum redundancy maximum relevance (mRMR), and recursive feature elimination (RFE). LASSO regression was performed with cross-validation to determine the optimal regularization parameter, and variables were selected according to their regression coefficients. The Boruta algorithm, based on random forest modeling, assessed feature importance by comparing actual variables with randomly permuted variables to identify stable predictors. The mRMR method ranked variables according to their relevance to outcomes and redundancy among features to identify variables with high information gain. The RFE method recursively removed low-importance features and used cross-validation to determine the optimal feature subset. A consensus-based feature selection strategy was adopted, and variables consistently identified by all four methods were considered final predictors. Based on this strategy, age, Modic changes, Pfirrmann grade, and APHC were selected as the final predictors for subsequent model development. These predictors were used to construct six machine learning models, including Decision Tree, support vector machine (SVM), artificial neural network (ANN), linear discriminant analysis (LDA), ExtraTrees and XGBoost for predicting postoperative adverse outcomes in patients with LDH undergoing UBE surgery. Hyperparameters of all six algorithms were optimized using GridSearchCV with five-fold cross-validation, and the area under the receiver operating characteristic curve (ROC-AUC) was used to identify the optimal parameter combination. The optimized models were subsequently evaluated in the independent validation cohort. Model performance was evaluated using multiple metrics, including the area under the receiver operating characteristic curve (AUC), accuracy, precision, sensitivity, specificity, F1 score, and decision curve analysis (DCA). The classification threshold was set at 0.5, and accuracy, precision, sensitivity, specificity, and F1 score were calculated accordingly. In addition, learning curves were analyzed to evaluate model fitting and stability in the training and validation cohorts. DCA was further performed to compare the clinical utility of different models and identify the optimal model from a clinical decision-making perspective.

### 2.3 Model representation and development of the online calculator

**Figure 2:**
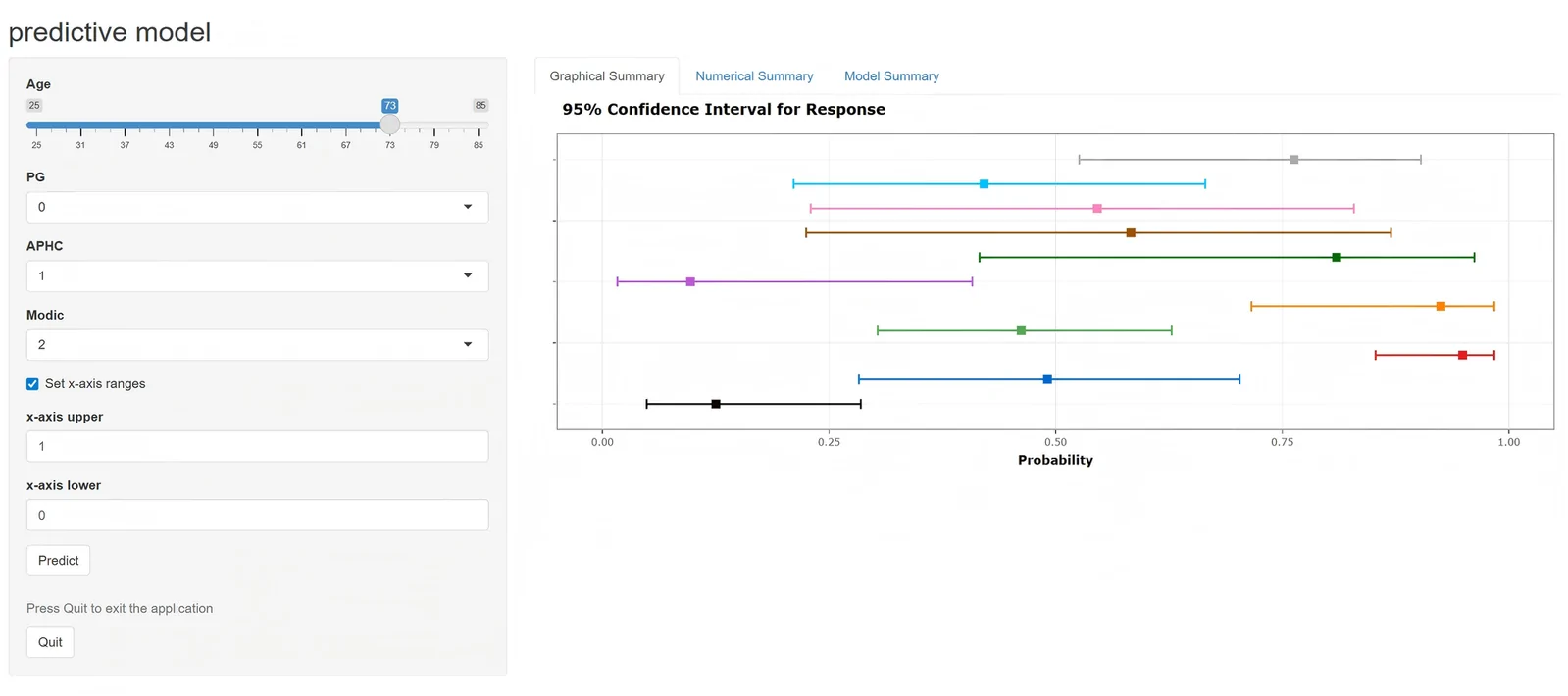
Development of an online calculator based on the extraTrees predictive model for estimating the risk of postoperative adverse outcomes in patients with LDH undergoing UBE surgery. PG, Pfirrmann grade; MC, Modic changes; APHC, facet joint hypertrophy and bony fusion.

An online calculator based on the prediction model was developed to assist clinicians in estimating the risk of adverse outcomes after UBE surgery for LDH using individual clinical and radiological characteristics. The web-based calculator was designed for convenient clinical application. The interface consists of three main sections.The first section, located on the left side of the interface, allows users to input model variables, including age, PG, MC, and APHC. After entering the required parameters, users can click the “Predict” button to obtain the estimated risk of adverse outcomes. The second section, located in the upper right quadrant of the interface, displays the risk factors associated with postoperative adverse outcomes. The contribution of each variable to the prediction is quantified according to its corresponding feature weight. The third section, labeled “Prediction Model,” records and summarizes previous prediction results for reference (Figure 2).

### 2.4 Statistical analysis

Descriptive statistics were used to summarize the baseline characteristics of the patients. The Shapiro–Wilk test was applied to assess the distributional normality of continuous variables. Normally distributed continuous variables were presented as mean ± standard deviation (SD), whereas non-normally distributed variables were presented as median (interquartile range, IQR). Between-group comparisons were performed using the independent-samples *t* test for normally distributed continuous variables and the Mann–Whitney U test for non-normally distributed variables. Categorical variables were summarized as frequencies and percentages and compared using the Pearson chi-square test or Fisher's exact test, as appropriate. Statistical analyses were conducted using SPSS Statistics version 27. Machine learning models were developed using Python version 3.10. A two-sided *P* value < 0.05 was considered statistically significant.

## 3 Results

### 3.1 Interobserver agreement analysis of radiological variables

To assess the reliability of radiological assessments, interobserver agreement for each imaging parameter was evaluated using the Kappa coefficient (Table 1). The results demonstrated substantial agreement among observers for all evaluated radiological parameters. The Kappa values were 0.877 (95% CI, 0.827–0.927) for HT, 0.912 (95% CI, 0.880–0.944) for PG, 0.901 (95% CI, 0.861–0.941) for MC, 0.875 (95% CI, 0.823–0.927) for APHC, 0.875 (95% CI, 0.826–0.923) for disc calcification, and 0.887 (95% CI, 0.845–0.929) for LHD. All Kappa analyses were statistically significant (all *P* < 0.001), indicating excellent interobserver agreement and high reliability of the radiological assessments.

**Table 1:** Interobserver agreement of radiological variables assessed by the kappa coefficient.

Variables	Kappa Value	95%CI		Z	*P*
		Lower	Upper		
HT	0.877	0.827	0.927	17.967	＜0.001
PG	0.912	0.880	0.944	25.768	＜0.001
Modic	0.901	0.861	0.941	23.732	＜0.001
APHC	0.875	0.823	0.927	17.917	＜0.001
Calcification	0.875	0.826	0.923	17.903	＜0.001
Level of herniated disc	0.887	0.845	0.929	23.230	＜0.001

### 3.2 Baseline characteristics

A total of 418 patients were included, with 292 assigned to the training cohort and 126 assigned to the validation cohort. In the overall cohort, 157 patients (37.6%) experienced adverse outcomes, whereas 261 patients (62.4%) had favorable outcomes. In the training cohort, adverse outcomes occurred in 111 patients (38.0%), whereas favorable outcomes were observed in 181 patients (62.0%). In the validation cohort, 46 patients (36.5%) experienced adverse outcomes, whereas 80 patients (63.5%) had favorable outcomes. The incidence of adverse outcomes was comparable between the training and validation cohorts, indicating similar outcome distributions between the two cohorts. No statistically significant differences were observed in baseline characteristics between the two cohorts (all *P* > 0.05; Table 2).

**Table 2:** Baseline characteristics of patients in the training and validation cohorts.

Variables	Total (*n* = 418)	Train set (*n* = 292)	validation set (*n* = 126)	Statistic	*P*
Age, Mean ± SD	68.82 ± 10.77	68.88 ± 10.92	68.70 ± 10.46	t = 0.16	0.875
BMI, Mean ± SD	26.42 ± 5.70	26.49 ± 5.79	26.26 ± 5.49	t = 0.39	0.697
Course of disease, Mean ± SD	13.59 ± 3.91	13.73 ± 3.83	13.27 ± 4.09	t = 1.09	0.277
Operation time, Mean ± SD	114.86 ± 15.73	115.00 ± 15.81	114.52 ± 15.59	t = 0.29	0.774
Gender, n(%)				*χ*^2^ = 1.93	0.165
Female	209 (50.00)	140 (47.78)	69 (55.20)		
Male	209 (50.00)	153 (52.22)	56 (44.80)		
PG, n(%)				χ^2^ = 8.10	0.088
Grade I	239 (57.18)	158 (53.92)	81 (64.80)		
Grade II	85 (20.33)	66 (22.53)	19 (15.20)		
Grade III	46 (11.00)	36 (12.29)	10 (8.00)		
Grade IV	33 (7.89)	25 (8.53)	8 (6.40)		
Grade V	15 (3.59)	8 (2.73)	7 (5.60)		
Level of herniated disc, n(%)				χ^2^ = 1.93	0.381
L3/4	36 (8.61)	27 (9.22)	9 (7.20)		
L4/5	254 (60.77)	182 (62.12)	72 (57.60)		
L5/S1	128 (30.62)	84 (28.67)	44 (35.20)		
Hypertension, n(%)				χ^2^ = 0.50	0.480
NO	250 (59.81)	172 (58.70)	78 (62.40)		
YES	168 (40.19)	121 (41.30)	47 (37.60)		
Osteoporosis, n(%)				χ^2^ = 0.16	0.686
NO	227 (54.31)	161 (54.95)	66 (52.80)		
YES	191 (45.69)	132 (45.05)	59 (47.20)		
ASA Classification, n(%)				χ^2^ = 0.17	0.921
I	161 (38.52)	111 (37.88)	50 (40.00)		
II	233 (55.74)	165 (56.31)	68 (54.40)		
III	24 (5.74)	17 (5.80)	7 (5.60)		
Diabetes, n(%)				χ^2^ = 0.02	0.876
NO	183 (43.78)	129 (44.03)	54 (43.20)		
YES	235 (56.22)	164 (55.97)	71 (56.80)		
Calcification, n(%)				χ^2^ = 0.57	0.451
NO	145 (34.69)	105 (35.84)	40 (32.00)		
YES	273 (65.31)	188 (64.16)	85 (68.00)		
Drink, n(%)				χ^2^ = 3.73	0.053
NO	177 (42.34)	133 (45.39)	44 (35.20)		
YES	241 (57.66)	160 (54.61)	81 (64.80)		
Smoke, n(%)				χ^2^ = 1.09	0.296
NO	201 (48.09)	136 (46.42)	65 (52.00)		
YES	217 (51.91)	157 (53.58)	60 (48.00)		
HT, n(%)				χ^2^ = 0.03	0.854
Central	123 (29.43)	87 (29.69)	36 (28.80)		
Lateral	295 (70.57)	206 (70.31)	89 (71.20)		
APHC, n(%)				χ^2^ = 0.01	0.905
NO	306 (73.21)	214 (73.04)	92 (73.60)		
YES	112 (26.79)	79 (26.96)	33 (26.40)		
Modic, n(%)				χ^2^ = 0.22	0.974
NO	281 (67.22)	198 (67.58)	83 (66.40)		
Type I	67 (16.03)	46 (15.70)	21 (16.80)		
Type II	41 (9.81)	28 (9.56)	13 (10.40)		
Type III	29 (6.94)	21 (7.17)	8 (6.40)		

### 3.3 Feature selection

**Figure 3:**
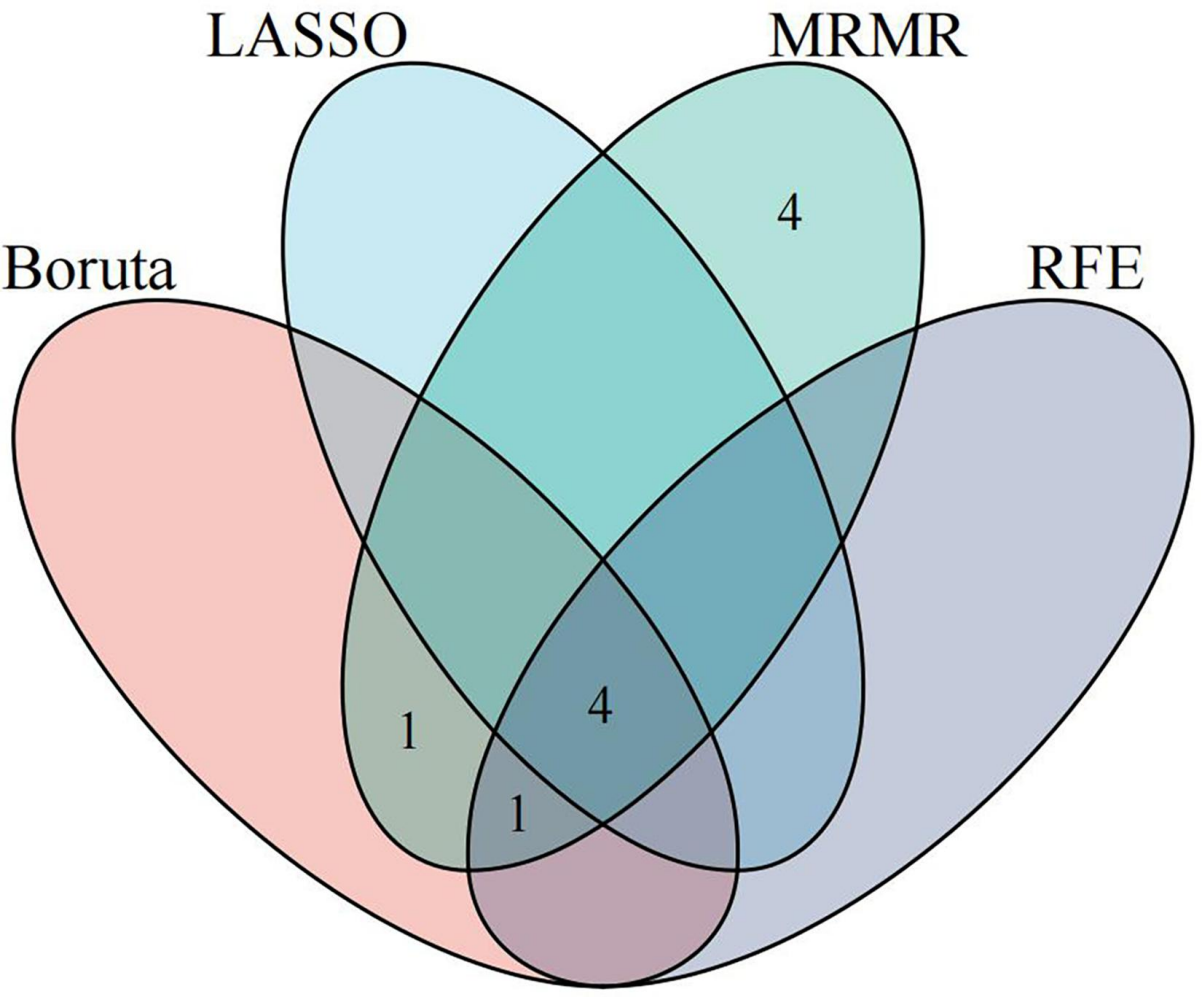
The four feature selection methods, namely boruta, lasso, RFE, and mRMR. LASSO, least absolute shrinkage and selection operator regression; Boruta, Boruta algorithm; MRMR, minimum redundancy maximum relevance; RFE, recursive feature elimination.

To identify variables associated with adverse outcomes after UBE, feature selection was performed using four methods: LASSO, Boruta, mRMR, and RFE. Figure 3 shows the overlap among the sets of variables selected by these four methods. Based on this overlap, four candidate predictors were retained: age, Modic changes, PG, and APHC (Supplementary Table S1).

### 3.4 Model performance and comparison

**Figure 4:**
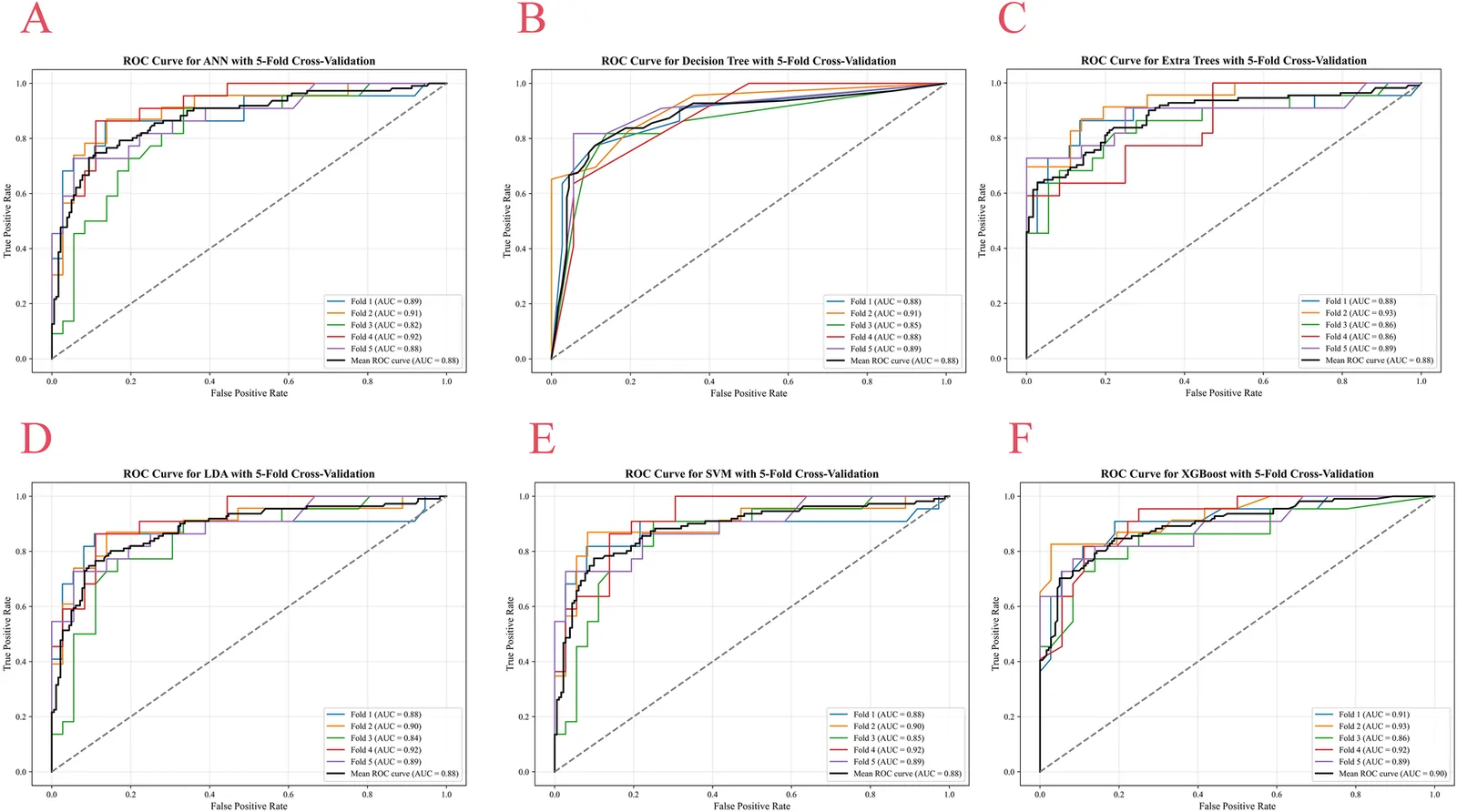
ROC curves of each machine learning model under 5–fold cross–validation: **(A)** artificial neural network (ANN); **(B)** decision tree; **(C)** extra trees; **(D)** linear discriminant analysis (LDA); **(E)** support vector machine (SVM); and **(F)** XGBoost.

**Figure 5:**
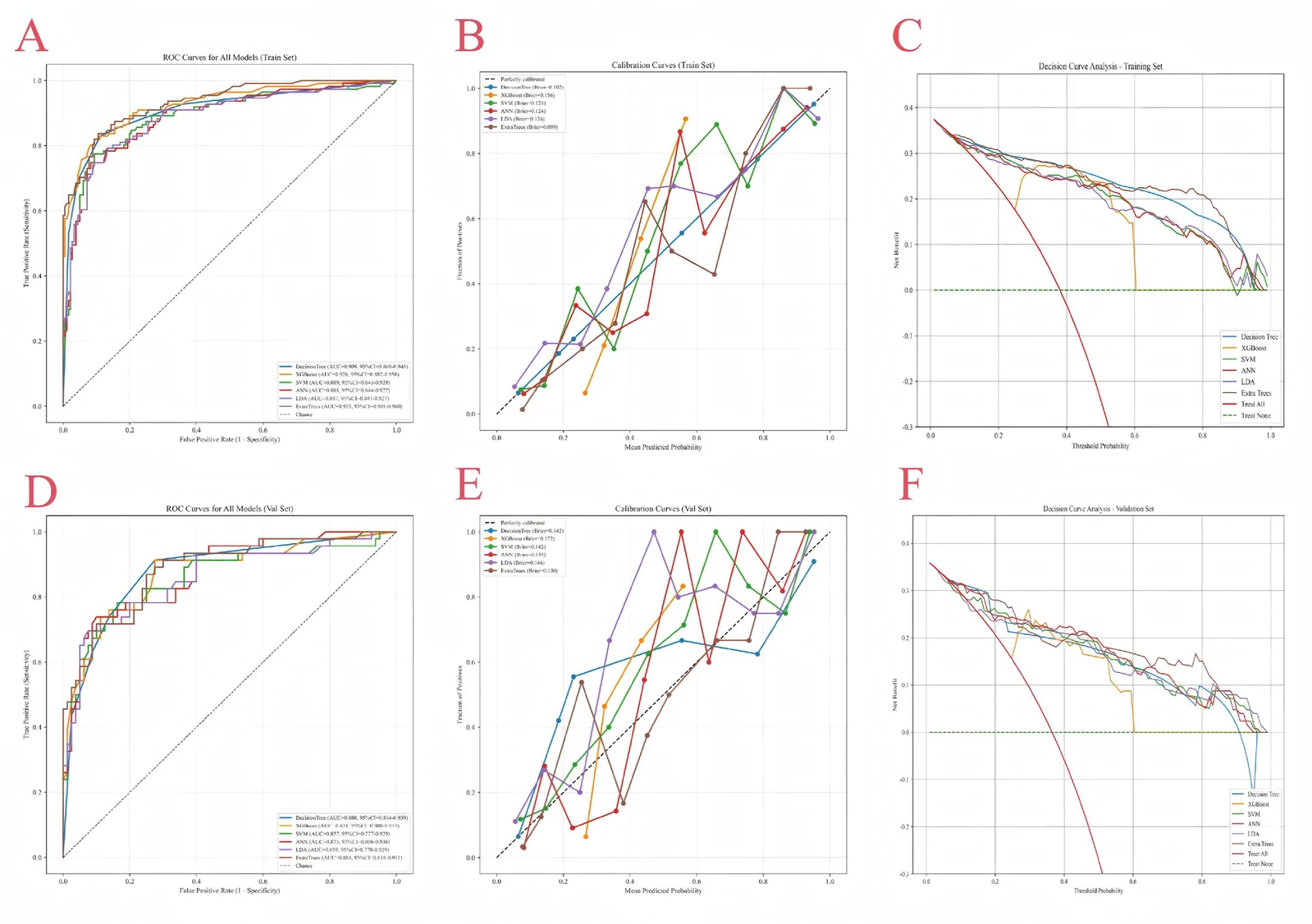
**(A)** receiver operating characteristic curve of the training set; **(B)** calibration curve of the training set; **(C)** decision curve analysis curve of the training set; **(D)** receiver operating characteristic curve of the validation set; **(E)** calibration curve of the validation set; **(F)** decision curve analysis curve of the validation set.

During model development, five-fold cross-validation was applied to the training set for model fitting and hyperparameter optimization, with the aim of improving model robustness and reducing the risk of overfitting (Figure 4). The predictive performance of the six machine learning models in the training and validation sets is summarized in Supplementary Table S2 and Supplementary Table S3, respectively.n the training set, ExtraTrees showed the highest discriminative performance, with an area under the receiver operating characteristic curve (AUC) of 0.9331, followed by XGBoost (AUC = 0.9265) and the decision tree model (AUC = 0.9086). The decision tree model achieved relatively high accuracy (0.8664), sensitivity (0.8288), and F1 score (0.8251), indicating comparatively strong performance in identifying patients with adverse outcomes in the training set. By contrast, XGBoost achieved the highest precision (0.9059) and specificity (0.9558), indicating a relatively low false-positive rate; however, its sensitivity was lower (0.6937). Among the remaining models, the artificial neural network (ANN) achieved an AUC of 0.8881, an accuracy of 0.8527, and an F1 score of 0.7943, reflecting relatively balanced performance across these metrics. The support vector machine (SVM) achieved high specificity (0.9392) but relatively low sensitivity (0.6757). Linear discriminant analysis (LDA) achieved an AUC of 0.8869, a specificity of 0.9282, and a sensitivity of 0.6667.In the validation set, ExtraTrees retained comparatively strong overall predictive performance. Its discrimination, calibration, and clinical net benefit were better than or comparable to those of the other models (Figure 5). Quantitative calibration analysis yielded Brier scores of 0.099 and 0.130 for ExtraTrees in the training and validation sets, respectively. The corresponding calibration intercepts were 0.301 and 0.235, and the calibration slopes were 1.550 and 1.194, respectively. In the validation set, the calibration intercept and slope were closer to their ideal values of 0 and 1, respectively. Together with the calibration curves, these estimates suggest that the model had acceptable overall calibration, although residual miscalibration remained. Nevertheless, the sensitivity of ExtraTrees in the validation set was 0.652, indicating that approximately 34.8% of patients with adverse outcomes were not identified as positive at the selected classification threshold. Therefore, if the model is used to screen for patients at high risk of adverse outcomes, the clinical consequences of false-negative predictions should be considered. The classification threshold may need to be adjusted according to the intended clinical use, particularly when higher sensitivity is prioritized. Lowering the classification threshold would generally increase sensitivity but would also increase the false-positive rate and reduce specificity. Thus, the threshold should be selected according to the relative clinical consequences of false-negative and false-positive classifications. Future studies should evaluate clinically appropriate risk thresholds in independent external validation cohorts and assess their utility in screening settings. Collectively, the ROC, calibration, and decision curve analyses indicated that ExtraTrees had the most favorable overall predictive performance among the six models evaluated. ExtraTrees was therefore selected as the final prediction model for subsequent analyses.

### 3.5 Model interpretation

**Figure 6:**
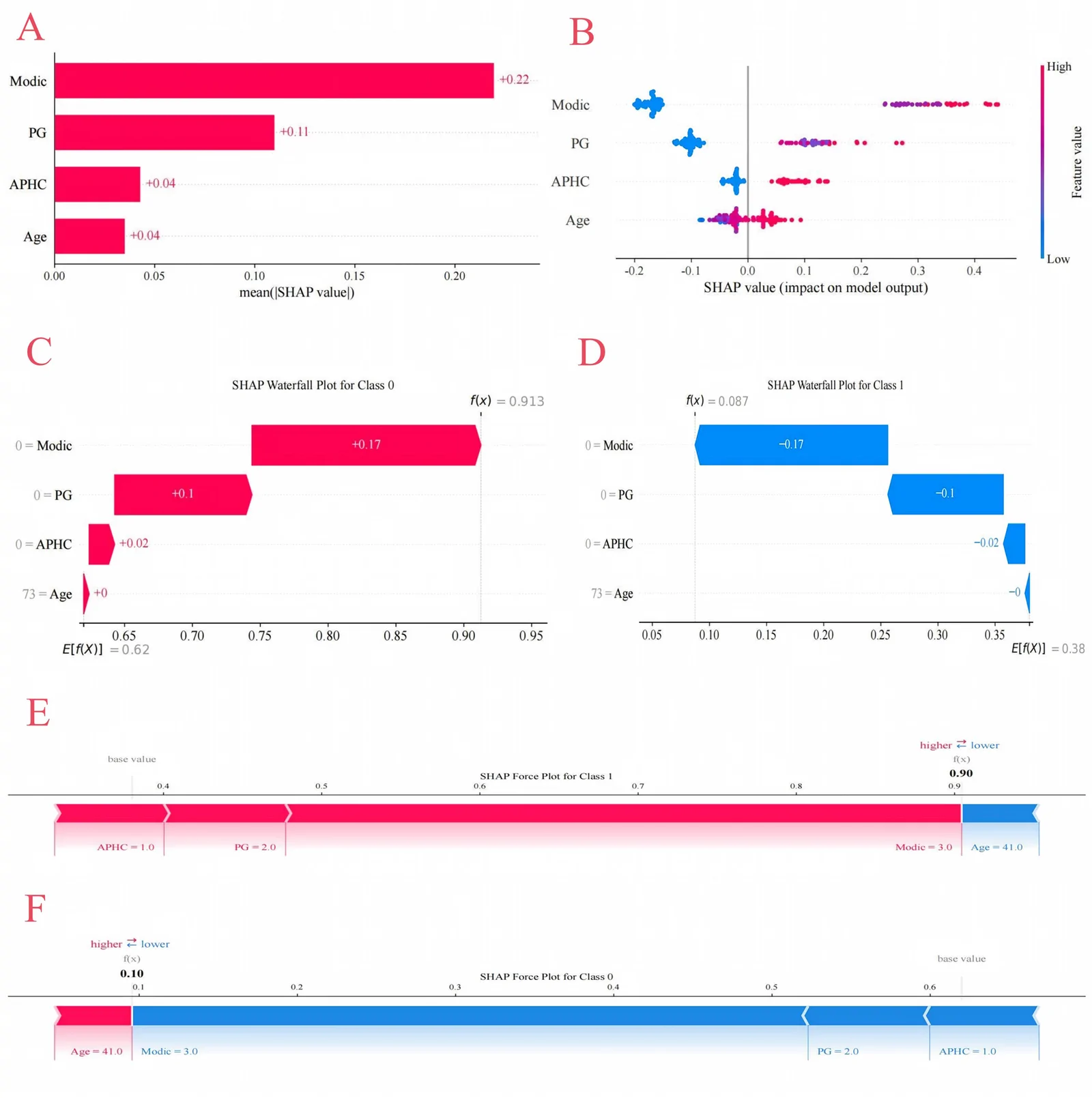
**(A)** SHAP feature importance bar plot showing the global feature importance of the extraTrees model for class 1 prediction. **(B)** SHAP summary plot illustrating the magnitude and direction of feature contributions to Class 1 prediction. **(C)** SHAP waterfall plot showing the individual contributions of features to a representative Class 0 prediction. **(D)** SHAP waterfall plot showing the individual contributions of features to a representative Class 1 prediction. **(E)** SHAP force plot illustrating the contribution of individual features to a Class 1 prediction. **(F)** SHAP force plot illustrating the contribution of individual features to a Class 0 prediction. Class 0 represents favorable outcomes, whereas Class 1 represents adverse outcomes; PG, Pfirrmann grade; MC, Modic changes; APHC, facet joint hypertrophy and bony fusion.

To further interpret the contribution of individual variables to model predictions, SHAP analysis was performed to evaluate feature importance at both the global and individual levels. As shown in the SHAP summary bar plot (Figure 6A), the mean absolute SHAP value was used to quantify the contribution of each feature to the model prediction. Features were ranked according to their mean absolute SHAP values, with age, PG, MC, and APHC identified as the four most important predictors. The SHAP summary dot plot (Figure 6B) provides a global visualization of feature effects in the ExtraTrees model, illustrating the direction and magnitude of each feature’s contribution to predictions. Higher MC and PG values (shown in red) were predominantly distributed in the positive SHAP value region, indicating that higher grades of these features increased the predicted probability of adverse outcomes. Conversely, lower MC and PG values (shown in blue) were mainly distributed in the negative SHAP value region, suggesting a lower contribution to adverse outcome prediction. The SHAP waterfall plot (Figure 6C) and SHAP force plot (Figure 6D) illustrate the contribution of individual features to the prediction for case 1. The specific value of each feature and its corresponding SHAP value indicate its positive or negative contribution to the final prediction.

### 3.6 LIME-Based local interpretation results

**Figure 7:**
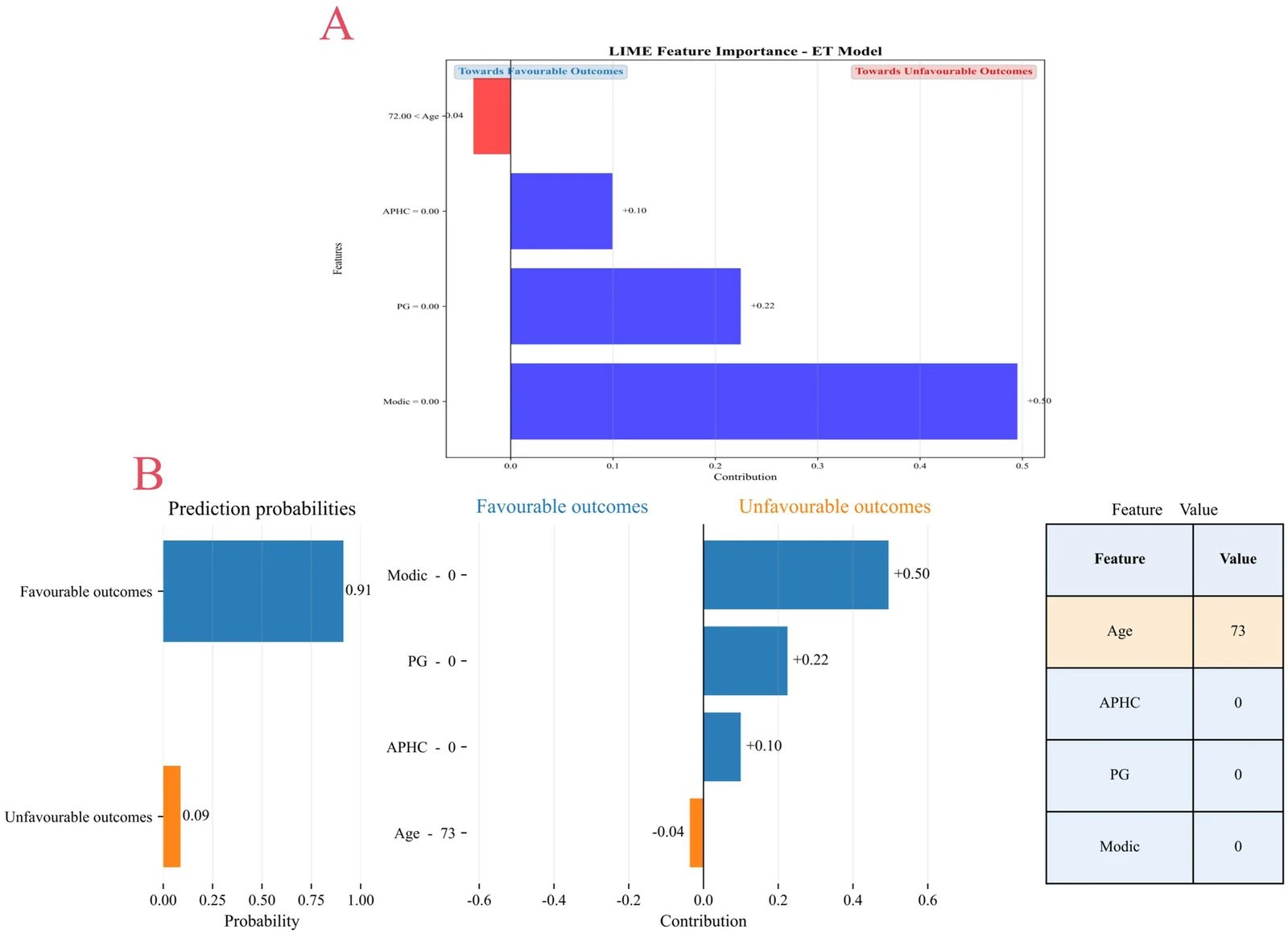
**(A)** LIME feature contribution plot for the extraTrees model; **(B)** LIME-based prediction explanation showing class probabilities and feature-wise contributions. PG, Pfirrmann grade; MC, Modic changes; APHC, facet joint hypertrophy and bony fusion.

Local interpretability analysis of the ExtraTrees model based on LIME is presented in Figure 7. Figure 7A illustrates the contribution of individual features to the model prediction in a representative case. Among all features, MC had the largest contribution to the model prediction (+0.50), followed by PG (+0.22) and APHC (+0.10). These features primarily shifted the prediction toward a favorable outcome. In contrast, age (>72 years) showed a mild negative contribution (−0.04), shifting the prediction toward an adverse outcome. Figure 7B further shows the predicted probability distribution for this case. The model predicted a 0.91 probability of a favorable outcome and a 0.09 probability of an adverse outcome. Although some features contributed negatively at the local level, the combined contributions of multiple features resulted in classification of this patient as having a favorable outcome. These findings indicate that the model prediction was not determined by a single feature but resulted from the combined contributions of multiple features.

## 4 Discussion

An interpretable machine-learning model was developed and validated to predict adverse outcomes after UBE for LDH using clinical and imaging features. Candidate predictors were screened using multiple feature-selection strategies, and four key factors were identified: age, MC, PG, and APHC. Among six algorithms, ExtraTrees showed the best overall performance. The model showed good discrimination and generalizability in the training and validation sets. Decision curve analysis indicated higher net benefit across most threshold probabilities. SHAP showed stable, directionally consistent contributions of the four predictors to the model output, supporting interpretability and clinical acceptability.

In this study, MC and PG were identified as major contributors to the predictive model, suggesting that degeneration of the intervertebral disc and vertebral endplate complex may represent important factors associated with adverse outcomes after UBE surgery. MC occurs more frequently among patients with lumbar degenerative diseases than in the general population (15). The development of MC may be associated with immune-mediated inflammatory responses triggered by exposure of nucleus pulposus tissue to the immune system. This inflammatory response may further impair endplate integrity and contribute to the initiation and progression of MC (16). Previous studies have suggested that fusion surgery may provide superior outcomes compared with decompression alone in selected LDH patients with MC (17), indicating that MC may represent not only a radiological finding but also a biologically active pathological condition associated with increased inflammation. Furthermore, disc calcification may impair intervertebral mobility and accelerate degeneration, while affecting adjacent endplate structures and contributing to the development or progression of MC (18). Previous studies have demonstrated that higher PG grades are associated with more advanced disc degeneration, reduced disc hydration, and more complex annular disruption and nucleus pulposus herniation patterns, which may contribute to increased surgical difficulty and complication risk (19). During UBE procedures, severely degenerated discs with advanced PG grades (IV–V) often exhibit increased stiffness and reduced elasticity, making endoscopic grasping and removal technically challenging. Additional instruments, such as shavers or high-speed burrs, may therefore be required, potentially prolonging operative time and increasing the risks of intraoperative bleeding and nerve root traction injury. Moreover, endplate sclerosis, vertebral osteophyte formation, and loss of disc height may further narrow the working space, complicating nerve root and osseous decompression and increasing the risk of intraoperative manipulation of the dural sac or nerve roots.

APHC was identified as another important predictor in this study. APHC reflects chronic degenerative changes of the facet joints, including facet hypertrophy, osteophyte formation, and bony bridging or fusion-like changes. These changes indicate substantial bony narrowing, increased anatomical complexity, and potential biomechanical abnormalities at the target segment, which may affect decompression adequacy and postoperative recovery through multiple mechanisms (20, 21). APHC frequently coexists with osseous narrowing of the spinal canal or lateral recess and may be accompanied by ligamentum flavum hypertrophy and facet joint capsule thickening. These changes may result in a combined pattern of nerve root compression caused by both disc herniation and bony stenosis. When decompression focuses primarily on removal of the herniated disc fragment or limited bony resection, residual lateral recess or nerve root canal stenosis may remain, resulting in incomplete neural decompression. Clinically, this may manifest as inadequate pain relief, residual numbness, or fluctuating symptoms, potentially compromising treatment effectiveness and patient satisfaction (22). Furthermore, posterior bony hypertrophy and irregular anatomy associated with APHC may substantially increase the difficulty of endoscopic visualization and precise control of the decompression boundary. Compared with isolated disc herniation, posterior column hypertrophy may result in limited working space, unclear bony landmarks, and unstable visualization, increasing the risk of incomplete decompression. Conversely, to minimize neural injury, surgeons may adopt conservative bony resection strategies, potentially resulting in residual compression. However, excessive facet joint resection intended to achieve sufficient decompression may compromise posterior structural stability, leading to persistent postoperative low back pain, altered segmental biomechanics, and potential instability (23). Achieving a balance between adequate decompression and preservation of spinal stability is particularly challenging in patients with prominent APHC, which may partly explain its association with adverse outcomes. In addition, APHC may reflect long-term degenerative changes within the affected segment, accompanied by alterations in the local biological environment and enhanced chronic inflammatory activity. These conditions may contribute to perineural fibrosis, adhesion around the dural sac and nerve roots, and impaired local microcirculation. During UBE procedures, such pathological changes may increase the difficulty of nerve root mobilization and the risk of neural manipulation. Even without apparent intraoperative complications, persistent nerve root edema or inflammatory responses may delay symptom resolution or contribute to residual symptoms and incomplete clinical improvement (24). The influence of APHC on surgical outcomes may be particularly pronounced during the early learning phase of UBE. UBE requires precise management of endoscopic visualization, planning of bony decompression extent, delicate manipulation of neural structures, and effective control of hemostasis and irrigation. When substantial posterior column degeneration is present, limited exposure, sclerotic bone, and irregular bony proliferation may further increase technical difficulty and the risks of incomplete decompression, intraoperative neural irritation, and residual compression (25, 26). Therefore, APHC may serve as a marker of anatomical complexity, reflecting degenerative burden and surgical difficulty. Its association with adverse postoperative outcomes may be amplified during the early learning curve, providing a plausible explanation for its high predictive contribution in the present model. Additionally, older age is frequently associated with paraspinal muscle fatty infiltration, sarcopenia, and multilevel degenerative changes, which may impair spinal biomechanical balance and increase the risk of postoperative complications and delayed recovery. These pathological factors were also reflected in the interpretability analysis, providing a clinically plausible pathophysiological basis for understanding the model predictions.

Several limitations should be considered when interpreting the findings of this study. First, this study used a single-center retrospective design. Patient selection, perioperative management, imaging assessment, and outcome adjudication were all conducted at the same institution. Therefore, selection bias, information bias, and residual confounding from unmeasured factors could not be fully excluded. Accordingly, the variables identified in this study should be interpreted as predictors of postoperative adverse outcomes rather than as causal determinants. Second, all procedures were performed by a single spine surgeon with extensive experience in UBE. This design reduced heterogeneity in surgical proficiency, decompression strategies, and operative workflows, thereby improving consistency during model development. However, the model may partly reflect surgeon-specific patient selection criteria, technical preferences, and perioperative management practices. At present, the model is most applicable to patients with lumbar disc herniation who meet the study eligibility criteria, undergo primary UBE, and are treated by experienced UBE surgeons using standardized operative protocols. Its predictive performance remains uncertain in patients undergoing revision surgery, in those with marked lumbar spinal stenosis or segmental instability, and in procedures performed by surgeons early in the UBE learning curve. Its performance also remains uncertain across different surgical teams and institutions. Third, internal validation was performed by randomly splitting the single-center cohort into training and validation sets. Although the validation set was not used for feature selection, model training, or hyperparameter optimization, the training and validation sets were drawn from the same institution, study period, surgeon, and clinical workflow. Therefore, this validation strategy primarily assesses model performance in data generated under similar conditions. It cannot substitute for temporal, geographic, or independent multicenter external validation. Differences among institutions in patient characteristics, disease spectrum, imaging interpretation, surgical techniques, and adverse-outcome incidence may affect model discrimination, calibration, and clinical net benefit. Accordingly, the model should not be implemented in other clinical settings until independent external validation has been completed and any necessary recalibration has been performed. Fourth, the prediction endpoint was defined as the postoperative outcome at 3 months. Consequently, the model primarily estimates the risk of early adverse outcomes, including inadequate pain relief, persistent or worsening symptoms, and short-term recurrence. It does not estimate the risks of delayed recurrence, segmental instability, persistent low back pain, reoperation, or poor long-term functional recovery. Therefore, the model should be interpreted only as a tool for estimating the risk of the composite adverse outcome within 3 months after surgery. It should not be used to predict long-term prognosis or reoperation risk. Its predictive performance and clinical utility at 6 months, 1 year, and longer follow-up intervals require further evaluation. Finally, the model was developed primarily from routinely collected clinical variables and preoperative imaging features. Several detailed anatomical and technical factors that may influence procedural complexity and postoperative recovery were not systematically included. These factors included the severity of lateral recess and foraminal stenosis, ligamentum flavum hypertrophy, nerve root adhesions, decompression extent, the proportion of facet joint resected, irrigation pressure, intraoperative blood loss, and intraoperative complications. The omission of these variables may have limited the model's ability to capture procedural complexity and postoperative risk comprehensively. Future studies should use prospective, multicenter designs involving multiple surgeons and longer follow-up. The model should be independently evaluated in temporally and geographically distinct cohorts to assess its discrimination, calibration, and clinical net benefit. Recalibration or model updating should be performed when necessary to maintain performance across institutions and surgeon populations.

## 5 Conclusion

The proposed prediction model demonstrated favorable predictive performance in the internal validation cohort and may serve as a practical tool for preoperative risk assessment of adverse outcomes following UBE surgery for LDH. This model may assist in identifying high-risk patients and provide evidence to support preoperative planning, patient counseling, and individualized treatment optimization. However, because validation was limited to a randomly divided single-center dataset, further external validation involving multicenter cohorts, different temporal periods, and surgeons with varying levels of experience is required to determine the model's robustness and generalizability.

## Data Availability

The raw data supporting the conclusions of this article will be made available by the authors, without undue reservation.
