# Supplementary material for: Development of a predictive model for adverse outcomes after unilateral biportal endoscopic spine surgery in the treatment of lumbar disc herniation using six interpretable machine learning models
Source: Front Surg. 2026 Aug 31;13:1908118. doi: 10.3389/fsurg.2026.1908118 (PMC13572689; doi:10.3389/fsurg.2026.1908118)
Supplement: Supplementary file 1 [file Table1.docx]

| **Feature-selection category** | **Number** | **elected features** |
| --- | --- | --- |
| **Boruta** | 6 | Age,PG,Hypertension,Calcification,APHC,Modic |
| **LASSO** | 4 | Age,PG,APHC,Modic |
| **MRMR** | 10 | Age,PG,Hypertension,Calcification,APHC,Modic,Operation time, BMI,ASA,Gender |
| **RFE** | 5 | Modic, PG, Age, Calcification, APHC |
| **Boruta:MRMR:RFE** | 1 | Calcification |
| **Boruta:MRMR** | 1 | Hypertension |
| **Boruta:LASSO:MRMR:RFE** | 4 | Modic, PG, Age, APHC |
| **Four features uniquely selected by mRMR** | 4 | ASA，BMI，Operation time，Gender |
