# Supplementary material for: Development of a predictive model for adverse outcomes after unilateral biportal endoscopic spine surgery in the treatment of lumbar disc herniation using six interpretable machine learning models
Source: Front Surg. 2026 Aug 31;13:1908118. doi: 10.3389/fsurg.2026.1908118 (PMC13572689; doi:10.3389/fsurg.2026.1908118)
Supplement: Supplementary file 2 [file Table2.docx]

| Model | AUC | Accuracy | Precision | Specificity | Sensitivity | F1 |
| --- | --- | --- | --- | --- | --- | --- |
| DecisionTree | 0.9086 | 0.8664 | 0.8214 | 0.8895 | 0.8288 | 0.8251 |
| XGBoost | 0.9265 | 0.8562 | 0.9059 | 0.9558 | 0.6937 | 0.7857 |
| SVM | 0.8888 | 0.839 | 0.8721 | 0.9392 | 0.6757 | 0.7614 |
| ANN | 0.8881 | 0.8527 | 0.8469 | 0.9171 | 0.7477 | 0.7943 |
| LDA | 0.8869 | 0.8288 | 0.8506 | 0.9282 | 0.6667 | 0.7475 |
| ExtraTrees | 0.9331 | 0.8459 | 0.8667 | 0.9337 | 0.7027 | 0.7761 |
