# Supplementary material for: Development of a predictive model for adverse outcomes after unilateral biportal endoscopic spine surgery in the treatment of lumbar disc herniation using six interpretable machine learning models
Source: Front Surg. 2026 Aug 31;13:1908118. doi: 10.3389/fsurg.2026.1908118 (PMC13572689; doi:10.3389/fsurg.2026.1908118)
Supplement: Supplementary file 3 [file Table3.docx]

| Model | AUC | Accuracy | Precision | Specificity | Sensitivity | F1 |
| --- | --- | --- | --- | --- | --- | --- |
| DecisionTree | 0.8798 | 0.8095 | 0.8056 | 0.9125 | 0.6304 | 0.7073 |
| XGBoost | 0.8738 | 0.7937 | 0.8333 | 0.9375 | 0.5435 | 0.6579 |
| SVM | 0.8573 | 0.8095 | 0.8667 | 0.95 | 0.5652 | 0.6842 |
| ANN | 0.8747 | 0.8254 | 0.875 | 0.95 | 0.6087 | 0.7179 |
| LDA | 0.8587 | 0.8016 | 0.8621 | 0.95 | 0.5435 | 0.6667 |
| ExtraTrees | 0.884 | 0.8175 | 0.8108 | 0.9125 | 0.6522 | 0.7229 |
